# Effects of pH and temperature on striped catfish Pangasianodon hypophthalmus juvenile: Data on growth performance and survival rate

**DOI:** 10.1016/j.dib.2023.109826

**Published:** 2023-11-19

**Authors:** Muhammad Yazed Abduh, Nor Izaty Asra Aswadi, Nur Mohamed Alli Husna, Sajdin Syazana, Nor Hakim Norazmi-Lokman

**Affiliations:** a Faculty of Fisheries and Food Sciences, Universiti Malaysia Terengganu, Kuala Terengganu, Terengganu, 21030, Malaysia; b Centre for Fisheries and Aquaculture, Institute for Marine and Antarctic Studies, University of Tasmania, Taroona, Tasmania 7053, Australia

**Keywords:** Aquaculture, Husbandry, Specific growth rate, Feed conversion ratio, Rearing water condition

## Abstract

The growth performance and survival rates of juvenile striped catfish, *Pangasianodon hypophthalmus*, reared at various levels of pH and temperature were described in this article. Two rearing trials were conducted separately for pH and temperature where both trials lasted for 35 days. One hundred and twenty juveniles (1.5 ± 0.23 g) were randomly stocked into 12 rectangle glass aquariums (*n* = 10 fish/tank; three replicates per treatment) with 100 L of water for each trial. The treatment consisted of four different pH level (7.5, 8.0, 8.5 and 9.0) and four different temperature level (26 °C, 28 °C, 30 °C and 32 °C). The survival of fish was counted at the end of experiments, and the weight of the juvenile was measured once a week. The quantification of feed intake was determined through the measurement of the residual weight of the feeds after the feeding process. Upon the conclusion of the experiment, the data pertaining to weight and feed intake were utilized to calculate the specific growth rate (SGR) and food conversion ratio (FCR) as indicators of growth performance. Additionally, the number of live fish was employed to ascertain the survival rate. The data obtained from the calculation of SGR, FCR and survival rate were next subjected to a normality test, one-way analysis of variance (ANOVA), and a Tukey post-hoc test. The information in this article will help in the business, experimental, and personal usage for *P. hypophthalmus* juveniles rearing process.

Specifications Table

**Table:** 

Subject	Aquatic science
Specific subject area	Aquaculture
Type of data	Graph
How the data were acquired	Weighing balance and ruler were used for weight and length measurement. Live and dead individuals were counted to calculate survival rate. Feed conversion rate were measured based on total feed consumption divided with fish body weight gain. IBM SPSS statistics version 25 (IBM, US) was used for the statistical analysis.
Data format	Raw Analyzed
Description of data collection	Data on growth performance: Body weight measurements were taken once weekly for five weeks. Feed consumption: At the end of the 35-day rearing trial, the amount of feed consumed (weight) and fish weight measurements were taken. Data on survival rates: The numbers of living fish were quantified at the end of the 35-day rearing trial.
Data source location	Institution: Faculty of Fisheries and Food Science City/Town/Region: Kuala Terengganu, Terengganu Country: Malaysia Latitude and longitude (and GPS coordinates, if possible) for collected samples/data: Freshwater Hatchery, Faculty of Fisheries and Food Science, Universiti Malaysia Terengganu: 5°24′36.2″N 103°05′20.2″E
Data accessibility	1. Within the article 2. Repository name: Mendeley Data Data identification number: DOI:10.17632/8rcyzcw4yt.1 Direct URL to data: https://data.mendeley.com/datasets/8rcyzcw4yt

## 1 Value of the Data


 

• *P. hypophthalmus* is a prominent species cultivated in the South East Asian region because to its rapid growth attributes and substantial commercial appeal. The provided data will be essential in facilitating the cultivation of young fish for many objectives, including commercial, scientific, and private applications.
• Data on growth and survival of *P. hypophthalmus* reared under different parameter condition are useful for farmers and breeders in selecting the best culture system.
• The data holds potential for utilisation by farmers and scientists in future studies aimed at enhancing fish culture practises to achieve higher output levels and better overall quality.

## 2 Data Description

**Fig. 1:**
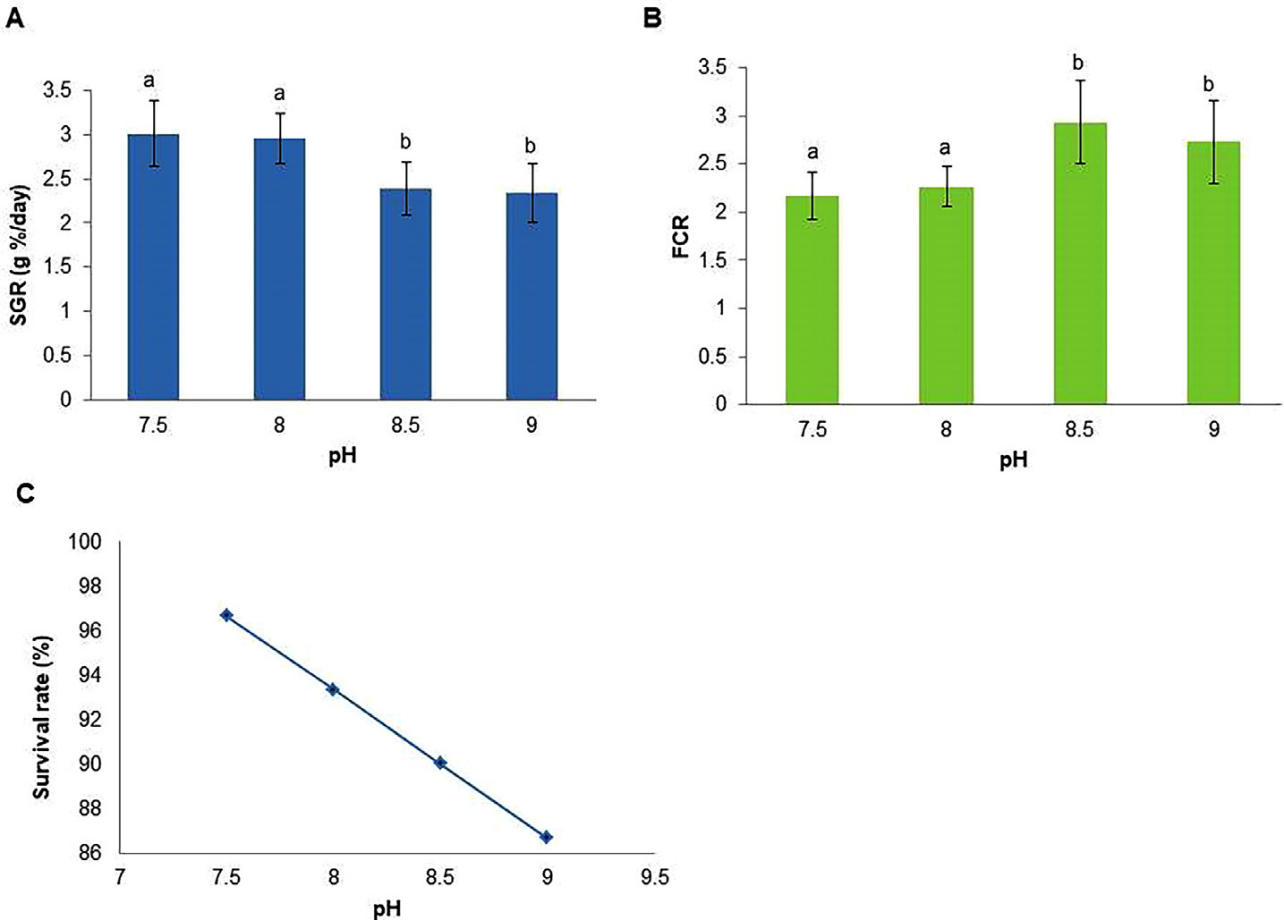
Specific growth rate (A), feed consumption ratio (B) and survival rate (C) of *Pangasianodon hypophthalmus* juveniles reared in different pH level. Different letters indicated significant (*p* < 0.05) difference.

**Fig. 2:**
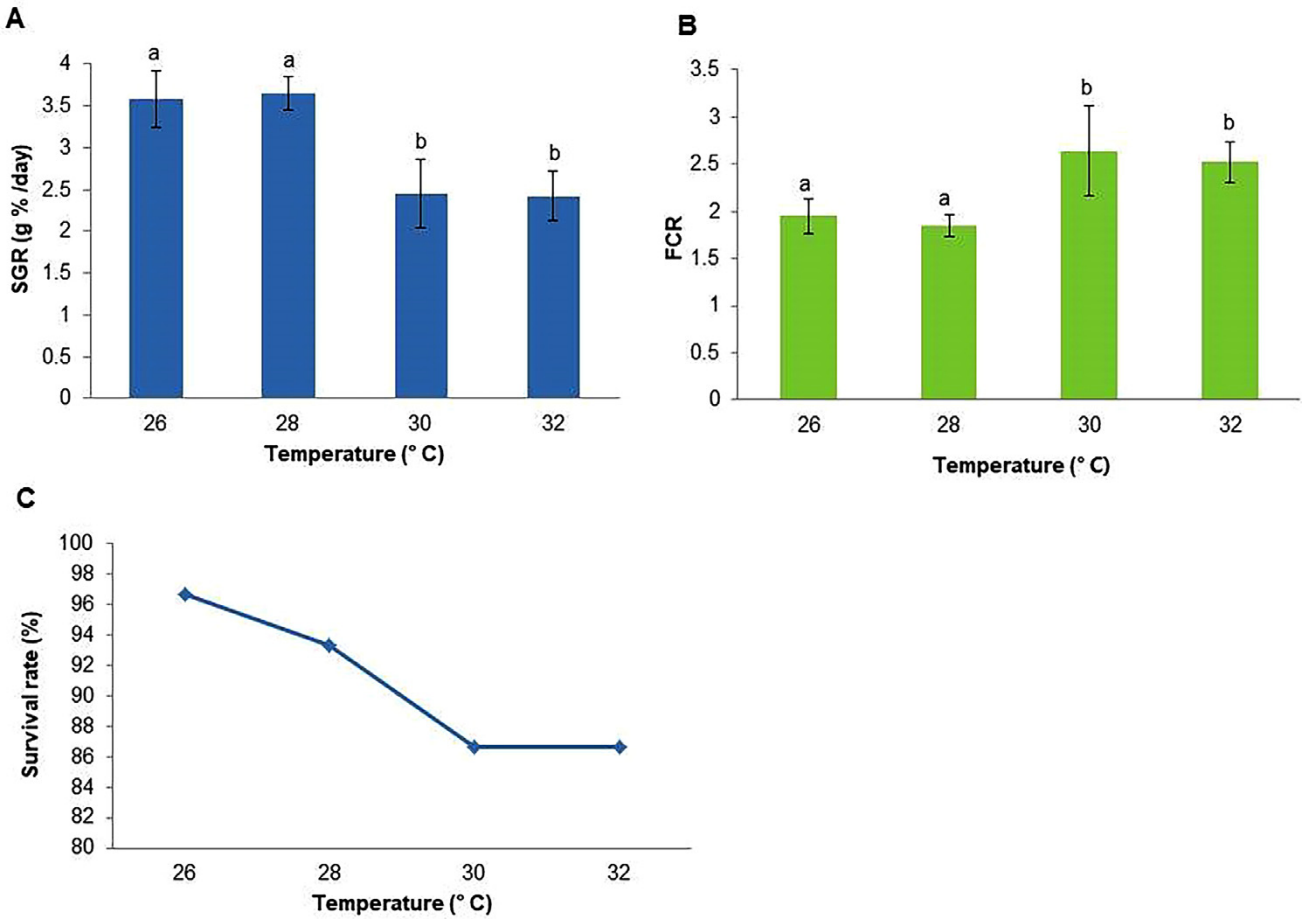
Specific growth rate (A), feed consumption ratio (B) and survival rate (C) of *Pangasianodon hypophthalmus* juveniles reared in different temperature. Different letters showed significant (*p* < 0.05) difference.

The raw data pertaining to fish weight, feed intake, and the number of living fish during the 35-day rearing trial may be found in [1]. The figures presented in Fig. 1, Fig. 2 depict the data pertaining to the specific growth rate (SGR), food conversion ratio (FCR), and survival rate for the two rearing trials conducted in this study. These figures were derived from calculations and analyses performed based on [1]. In the figures, A described the percentage of specific growth rate, B represented the feed consumption ratio and C showed the percentage of survival rate of *Pangasianodon hypophthalmus*. All data presented are as mean ± standard deviation.

## 3 Experimental Design, Materials and Methods

### 3.1 Location of Rearing Trial

The rearing trials were conducted at the Freshwater Hatchery, Faculty of Fisheries and Food Science, Universiti Malaysia Terengganu.

### 3.2 Fish Source and Maintenance

Two hundred and forty fishes were bought from a local commercial fish farm in Kuala Terengganu. The sex of the fish cannot be determined due to its small size (average weight of 1.54 g). The fishes were maintained in aquarium for ten days before the trials started. During the acclimation period, fish were fed twice a day up to satiation with commercial starter feed (Dinding, 32 % protein, 5 % lipid).

### 3.3 Experimental Design

In each trial, 12 glass aquariums (75 cm × 45 cm × 45 cm) were used, and each tank were stocked randomly with 10 juveniles (average length and weight of 5.0 cm and 1.54 g, respectively). Juveniles were provided with continuous aeration and were fed every day (2 times per day) where all faeces and feed residues were siphoned out.

#### 3.3.1 First Trial- Effects of Alkaline pH

The fish were exposed to four pH conditions which are 7.5 (control), 8.0, 8.5, 9.0 with three replicates per treatments [2]. Sodium hydroxide or ammonium hydroxide (0.01 M) was added to the experimental water until reached the desired pH level [2]. The pH levels were measured by using pH meter. Water exchanged were done every twice a week with 10 % of water that has been adjusted before. Due to the COVID 19 pandemics and the Movement Control Order by the government of Malaysia, the experimental period lasted for 35 days.

#### 3.3.2 Second Trial- Effects of Elevated Temperature

Four temperature conditions which are 26 (control), 28, 30 and 32 °C with three replicates per treatments were applied to the juveniles, following study by Islam et al. [3] with minor modification where different temperature control and the experiment duration were applied. Water heater was equipped in each treatment tank to maintain the required temperature. The rearing trial lasted also lasted for 35 days due to COVID 19 pandemics movement restrictions.

### 3.4 Data Analysis

For both rearing trials, number of fishes was counted and their weight was recorded at the starting (day 0) and final (day 35) of the experiments. A total of nine fish from each trial were collected with three replicates per treatments. The survival rate, feed conversion rate (FCR), specific growth rate (SGR) and weight gain was calculated by following the formula by Islam et al. [3] and Luo et al. [4]:

Survival rate = number of fish at the end of the experiment / number of fish at beginning of experiment x 100

Feed conversion ratio (FCR) = feed given (dry weight) / body weight gain (wet weight)

Specific growth rate (SGR, % day ^−1^) = 100 x (LnW_t_ – LnW_0_) /t

Where W_t_ = Final weight; W_0_ = Initial weight and t= trial duration (35 day)

### 3.5 Statistical Analysis

The data was analyzed using IBM SPSS Statistical Analysis programme (version 25). The normality of the data was assessed using the Shapiro-Wilk test prior to conducting a one-way ANOVA analysis. Subsequently, a Tukey post-hoc test was employed to ascertain any discrepancies in growth performances, feed intake, and survival rate between the two trials [5]. All value with P < 0.05, differences were deemed to be significant. All data were presented as mean ± standard deviation.

## Limitations

Not applicable.

## Ethics Statements

The authors affirm that all experiments conducted in this study adhere to the ARRIVE guidelines and were performed in accordance with the U.K. Animals (Scientific Procedures) Act, 1986 and its associated guidelines, EU Directive 2010/63/EU for animal experiments, or the National Institutes of Health guide for the care and use of Laboratory animals (NIH Publications No. 8023, revised 1978).

The study followed ethical and moral guidelines, which encompassed the appropriate treatment of animals and the determination of the minimal sample size necessary for statistically sound analysis. The aforementioned practices adhered to the Research Ethics Guidelines of Universiti Malaysia Terengganu.

## CRediT authorship contribution statement

**Muhammad Yazed Abduh:** Supervision, Conceptualization, Methodology, Validation, Resources, Writing – original draft, Writing – review & editing. **Nor Izaty Asra Aswadi:** Data curation, Formal analysis, Writing – review & editing. **Nur Mohamed Alli Husna:** Methodology, Investigation, Data curation, Formal analysis. **Sajdin Syazana:** Methodology, Investigation, Data curation, Formal analysis. **Nor Hakim Norazmi-Lokman:** Conceptualization, Formal analysis, Writing – original draft, Writing – review & editing.

## Data Availability

Raw data on effects of pH and temperature on Pangasianodon hypophthalmus juveniles (Original data) (Mendeley Data)
